# Apolipoprotein E synthesized by adipocyte and apolipoprotein E carried on lipoproteins modulate adipocyte triglyceride content

**DOI:** 10.1186/1476-511X-13-136

**Published:** 2014-08-23

**Authors:** Yan-hong Li, Ling Liu

**Affiliations:** Department of Cardiology, the Second Xiangya Hospital, Central South University, #139 Middle Renmin Road, Changsha, Hunan 410011 PR China

**Keywords:** Apolipoprotein E, Adipocyte, Triglyceride, Receptor-mediated endocytosis

## Abstract

Excessive energy storage of adipose tissue makes contribution to the occurrence and progression of obesity, which accompanies with multiple adverse complications, such as metabolic syndrome, cardiovascular diseases. It is well known that apolipoprotein E, as a component of lipoproteins, performs a key role in maintaining plasma lipoproteins homeostasis. Interestingly, apolipoprotein E is highly expressed in adipocyte and has positive relation with body fat mass. Apolipoprotein E knock-out mice show small fat mass compared to wild type mice. Moreover, adipocyte deficiency in apolipoprotein E shows impaired lipoproeteins internalization and triglyceride accumulation. Apolipopreotein E-deficient lipoproteins can not induce preadipocyte to form round full-lipid adipocyte, whereas apolipoprotein E-containing lipoproteins can. This article mainly reviews the modulation of apolipoprotein E synthesized by adipocyte and apolipoprotein E carried on lipoproteins in adipocyte triglyceride content.

## Introduction

During recent decades, obesity has reached epidemic proportions globally in developed and developing countries [1]. A person, with body mass index ≥30 kg/m^2^ or waist-hip ratio ≥102 cm for men and ≥88 for women, is defined as obesity according to the World Health Organization [2]. What is becoming increasingly evident is that obesity has become an independent risk of life-threatening diseases, such as insulin resistance, diabetes, cardiovascular diseases, stroke and certain cancers [3–6]. Those diseases have high morbidity and mortality, laying a huge economic burden on the society and the patient family. In addition, with the change of social acceptance and socio-cultural factors, overweight and obese individuals might suffer from psychological distress, for example, self-depreciation and depression [7]. Physical and psychological disorders induced by obesity have become increasingly serious public health problems. Therefore, it is worthy to better understand adipocyte and adipose tissue physiology.

Obesity is characterized with excessive adipose tissue mass. Adipose tissue can greatly expand its energy-buffering capacity by adipocyte hypertrophy and/or by adipocyte hyperplasia [8]. Adipocyte hypertrophy is defined as expanding in adipocyte size resulting from uptake fatty acids (FFA) and storage in form of triglyceride (TG). Adipocyte hyperplasia, also named adipogenesis, is termed as increase in adipocyte number, resulting from proliferation of undifferentiated fibroblast-like adipocyte progenitors and then differentiation into mature round lipid-filled adipocyte [9]. Adipocyte hypertrophy often precedes adipocyte hyperplasia in humans and animals [9].

Apolipoprotein E (ApoE), molecular mass of 34.5 kDa, is a glycoprotein coded by 299 amino acids. Due to amino acids rich in arginine, ApoE is also named as “arginine-rich” apolipoprotein [10], which is synthesized mainly by liver [11]. Other tissues and cells, like brain, kidney, adipocyte, smooth muscle cells and macrophages, also can produce ApoE [12, 13]. As a component of lipoproteins, ApoE mediates lipoproteins internalization and degradation via receptor-mediated endocytosis pathways. Recently, numerous studies have demonstrated that strong positive correlations exist between ApoE and triglyceride accumulation in adipocyte. ApoE synthesized by adipocyte is associated with adipocyte hypertrophy in vivo and in vitro [14–16]. ApoE carried on circulating lipoproteins is essential in lipoproteins-induced adipogenesis [17]. ApoE has three isoforms in humans, however, the relationship between ApoE polymorphism and body fat mass is still controversial [18–20].

### Apolipoprotein E

ApoE is a major component of very low density lipoproteins (VLDL), remnant lipoproteins (RLPs) and high density lipoproteins (HDL). In the early 1970s, ApoE was firstly discovered as a protein component of VLDL [21]. VLDL from live and chylomicron from intestine are termed as triglyceride-rich lipoproteins (TRLs). TRLs become enriched in ApoE as they are lipolyzed on the surface of endothelial cells by lipoprotein lipase (LPL) and convert into RLPs [22, 23]. Moreover, ApoE recycles among lipoproteins particles. TRLs with the higher content in ApoE provide material for ApoE and particle recycling. Once internalized, the majority of TRLs-derived ApoE remains in early endosomes. Upon stimulation with HDL or HDL-derived apolipoprotein A-I, ApoE can be recycled back to the plasma membrane, followed by ApoE re-secretion and the subsequent formation of ApoE-containing HDL. The HDL-induced recycling of ApoE may drive cholesterol efflux or capture new remnants [24, 25].

ApoE on the surface of lipoproteins is served as a natural ligand of receptors, which is essential in the metabolism of lipoproteins. Associated receptors includes low density lipoproteins receptor (LDLr), LDL receptor-related protein (LRP), VLDL receptor (VLDLr), heparin sulfate proteoglycans (HSPGs) [22, 26, 27]. These receptors are also termed as ApoE-recognizing receptors. LDLr, VLDLr and LRP belong to LDL receptor family, which bind ApoE in clathrin-dependent pathway [26] and preferentially clear a subset of large TRLs particles enriched in ApoE [28]. However, as specific HSPGs, syndecan-1 mediates lipoproteins endocytosis in lipid raft-dependent fashion [29] and preferentially clear small particles enriched in ApoE and apolipoprotein AV [28]. In addition, ApoE-containing HDL participate in cholesterol efflux and contribute to the maintenance of plasma and tissue cholesterol homeostasis [22, 23, 30].

Interestingly, ApoE is polymorphic and its function shows isoforms specificity in humans. There are three alleles, ϵ2, ϵ3 and ϵ4, and encoding there isforms ApoE2, ApoE3 and ApoE4, respectively [31]. The ApoE3 isoform is the most common genotype, accounting for about 78%. Recently, the rare haplotype has been reported in several subjects. This fourth ApoE allele is similar to ϵ3, and termed as the ϵ3r allele that encodes the ApoE3r isoform [31, 32]. In the lipid-binding function, ApoE3 and ApoE2 preferentially bind the smaller, highly curved lipid emulsions (e.g., HDL), while ApoE4 prefers larger, less curved lipid emulsions (i.e., VLDL). ApoE2 is weaker than ApoE3 and ApoE4 in the receptor-binding capacity. However, the heparin-binding function do not seem to be any major difference among there isoforms [22].

Clinically, the ApoE2 carriers have elevated plasma TG level and are prone to developing type III dyslipoproteinemia, which increase the risk for metabolic-associated diseases [33, 34]. The ApoE4 carriers have higher risk for cardiovascular disease, which attributed to higher concentrations of LDL-cholesterol [35]. Emerging studies showed that the impact of ApoE2 and ApoE4 on the cardiovascular disease risk phenotype was influenced by lipid fractions, inflammatory profile and adiposity in humans [36, 37]. Due to the very rarity of the ApoE3r isoform, its effect on lipoproteins metabolism remains unknown, and requires further investigations.

### The relationship between ApoE and body fat mass

It is well known that LPL is the rate-limiting enzyme for the hydrolysis of TG in core of TRLs and deficiency of this enzyme induces severe hypertriglyceridemia in humans and mice [38, 39]. Adipose fat storage is thought to require uptake of circulating TG-derived fatty acids mediated by LPL. However, humans with LPL deficiency and adipose-specific LPL deficient mice showed normal body fat mass [40, 41]. Recent report showed that hypertriglyceridaemia induced by the loss of LpL was associated with reduced TG uptake into brown adipose tissue, but white adipose tissue fat accumulation was normal [42]. In adipose tissue of LPL deficiency, chemical composition revealed that lipid storage maintained mainly by enhanced endogenous adipocte fatty acid synthesis [39, 41]. Increased de novo adipocyte lipogenesis could compensate for adipose LPL deficiency and play an important role in body fat mass. Therefore, it could conjecture that LPL-independent pathways contribute to adipocyte TG storage.

One possible LPL-independent pathway is receptor-mediated endocytosis in obesity. It was demonstrated that Apolipoprotein C-I specifically inhibited VLDL binding LDL receptor family [43]. Interestingly, human apolipoprotein C-I transgenic mice were protected from obesity [44]. Moreover, mice knocking out VLDLr or LRP displayed lower body weight compared with wild type (WT) controls [45, 46]. VLDLr-deficient patients were also underweight, with body mass index less than 18.5 [47]. ApoE is one of important ligands for these receptors binding lipoproteins. It seems reasonable to speculate that the receptor-mediated pathways associated with ApoE could perform a potential role in body fat mass.

The crucial role of ApoE in body fat mass has been clearly demonstrated in ApoE knock-out (EKO) mice. EKO mice feeding on a chow diet or high-fat diet were more resistant to the development of obesity than WT controls. Deletion of ApoE in genetic mutation obese mice, such as leptin deficient mice and Ay^/+^ mice, also prevented diet-induced obesity by either a high-cholesterol diet or high-sucrose diabetogenic diet [17, 48, 49]. EKO adipocyte showed low-level TG, low FFA content and limited lipoproteins uptake than WT controls [14–16]. However, the levels of TG and FFA doubled in cultured EKO adipocyte overexpressing adenoviral-mediated ApoE3 [14].

In humans, there is no established link between the loss of ApoE and body fat mass due to extremely rare ApoE-deficiency condition. It is well known ApoE is essential in the catabolism of lipoprorteins. Although EKO mice could prevent diet-induced obesity, it spontaneously develops severe hypercholesterolemia and atherosclerotic lesions that are similar to those found in humans [50, 51]. EKO mice is the most ideal genetically modified animal in atherosclerosis research. Therefore, the lack of functional ApoE have severe metabolic disorders, and the extremely rare ApoE-deficiency condition may be in accordance with the favourable selective pressure in humans.

However, it is also controversial about the relationship between ApoE polymorphism and obesity in different race. According to population epidemiological statistics, the ApoE3 isoform is the most common genotype and considered as wild type. A study showed that ApoE2 was a strong indicator for human obesity in the Roma and Turkish subjects [18, 52]. However, the Tehran Lipid and Glucose Study did not show any relation between ApoE isoforms and obesity in a Tehranian population [19]. ApoE genotype in obese population showed no significant statistical difference compared with that of lean controls in Chinese population of Chengdu area [20]. Actually, it is hard to definitely evaluate ApoE polymorphism in obesity on account of almost unavailable ApoE2 and ApoE4 homozygous in humans.

Interestingly, the effect of ApoE polymorphism in body fat mass existed significant difference in mice. ApoE2 knock-in mice were to easily develop hepertriglycerdemia and diet-induced obesity [53, 54]. However, ApoE4 knock-in mice presented a lower body mass index and reduction of adipogenesis after feeding high-fat Western-type diet [55]. The difference between mice and humans could be explained by different constituent and metabolism of lipoproteins. About 70% VLDL from mice contain apolipoprotein B48 particles, whereas all VLDL from humans contain apolipoprotein B100 particles [54]. Apolipoprotein B48 is a truncated form of apolipoprotein B, which does not contain the carboxyl-terminal portion of apo B and can not bind to LDL receptors. However, apolipoprotein B100 can be recognized by LDL receptor. Thus, mice depend more on apoE for VLDL clearance than humans do. Therefore, results from ApoE transgenic mice cannot be extrapolated to humans without taking this significant difference into consideration.

### ApoE synthesized by adipocyte in adipocyte triglyceride accumulation

More than two decades ago, Zechner et al. [13] firstly observed that adipocyte expressed high abundance of ApoE. Interestingly, adipocyte-derived ApoE expression was exactly paralleled with the development of visible lipid droplets in a differentiation-dependent fashion in vitro [13, 56]. It has been widely established that peroxisome proliferator-activated receptor gramme (PPARγ) is an essential regulator of adipogenesis [57]. More interestingly, PPARγ agonists increased the expression of adipocyte ApoE in vitro and in vivo [58]. Addition PPARγ agonists (i.e.rosiglitazone) to WT adipocyte led to TG and FFA accumulation, accompanying with a fourfold increase in adipocyte ApoE expression. However, those effects were blunted in EKO mice [14]. It could indicate that ApoE synthesized by adipocyte may have relation with adipocyte triglyceride accumulation.

ApoE synthesized by adipocyte performed an indispensable role in adipocyte triglyceride accumulation. In order to discriminate the potential effects of various derived ApoE in adipocyte triglyceride accumulation, Huang et al. [15] transplanted epididymal white adipose tissue from EKO or age- and gender-matched WT donors into WT recipients, respectively, to form EKO-WT and WT-WT models in vivo. After feeding either chow diet or high-fat diet for 8 ~ 10 weeks, transplanted white adipose tissue was harvested. EKO-WT adipocyte displayed smaller size, lower TG content, higher TG hydrolysis and lower TG synthesis than WT–WT controls. This difference was not because of absence of ApoE carried on circulating lipoproteins but ApoE derived from adipocyte. Thus, ApoE carried on lipoproteins could not correct the deficiency in triglyceride accumulation of EKO adipocyte.

There were two possible mechanisms to explain the impaired lipid storage in EKO adipocyte. On one hand, caveolin-1 (cav-1) were significantly suppressed in EKO adipocyte, which led to the impairment of both transportation of FFA across the adipocyte membrane and subsequent adipocyte triglyceride synthesis [14, 16, 48, 59]. This defect could be corrected by increasing cav-1 expression using viral transduction in cultured EKO adipocytes [16]. Cav-1 was a major functional protein of caveolae that were specialized membrane invaginations accounting for a third of adipocyte plasma membrane [60, 61]. During adipogenesis of 3 T3-L1 cells, a widely used model system for studying adipogenesis, cav-1 was dramatically induced as compared with the undifferentiated fibroblastic state [60]. Cav-1 null mice were overtly resistant to diet-induced obesity, lying in an inability to lipid storage in adipocyte [62]. Interestingly, adipocyte ApoE was colocalized with cav-1 at the adipocyte plasma membrane [63]. However, more detailed functional data will be needed to further elucidate the interaction between adipocyte ApoE and cav-1 in cellular lipid storage.

On the other hand, EKO adipocyte showed impaired receptor-mediated endocytosis. The expressions of ApoE-recognizing receptors were reduced in EKO adipocyte, or these receptors, such as VLDLr and LRP, redistributed away from the cell membrane surface [16]. It have reported that VLDLr were strongly induced during 3 T3-L1 adipogenesis [64]. Moreover, VLDLr deficiency protected mice from obesity [45]. The consistent results were observed in VLDLr-deficient subjects in humans. VLDLr-deficient patients had abnormally low body mass index (less than 18.5) compared with control subjects [47]. The increased expression of LRP was associated with proportionally increased endocytic activity in 3 T3-L1 adipocyte [65]. And mice with adipocyte-specific deletion LRP displayed smaller body fat than WT controls [46]. Thus, receptor-mediated endocytosis could promote adipocyte TG accumulation.

### ApoE carried on lipoproteins in adipocyte triglyceride accumulation

Circulation fatty acids that are available for adipocyte uptake are either hydrolysis product from TRLs by LPL, or in the form of TRLs. TRLs provides a concentrated source of esterified fatty acids, which could be directly internalized by adipocyte endocytic receptors, for example VLDLr, LRP, LDLr [16, 45, 46]. These receptors transport internalized lipoproteins to lysosomes for degradation, and producing intracellular FFA is assimilated into TG-rich storage droplets. It has been well clarified that these endocytic receptors via recognizing ApoE perform a vital role in hepatic clearance of TRLs and their remnant [28]. However, it deserves to explore whether or not ApoE takes part in receptor–mediated endocytic pathways in adipocyte triglyceride accumulation.

Tsuyoshi Chiba et al. [17] firstly demonstrated that ApoE carried on lipoproteins was essential for VLDL-induced adipogenesis. VLDL from WT mice induced the differentiation of bone marrow stromal cells to mature adipocyte with large lipid droplets, whereas ApoE-deficient VLDL from EKO mice failed. Similarly, selective removal of ApoE from human VLDL via trypsin treatment impaired the adipogenic effect of human VLDL. And preincubation of ApoE-deficient VLDL with recombinant human ApoE partially recovered the adipogenic activity.

The most possible mechanism of VLDL-induced adipogenesis was that adipocyte internalized VLDL particles via ApoE receptors [17]. This viewpoint is consistent with a previous study that ApoE at the surface of lipoproteins was required in receptor-mediated endocytosis in mice and humans [66]. EKO mice were more resistant to gaining body fat than LDLr deficiency mice, suggesting ApoE-dependent receptors, for example LRP, VLDLr, HSPG, could take part in adipocyte triglyceride accumulation [23]. However, it remains to be clarified which receptor has a stronger influence on adipocyte triglyceride accumulation.

HSPGs were independent of LDLr family members in clearance of both intestinally derived and hepatic lipoprotein particles [67]. It also showed that HSPGs increased in differentiated adipocyte and contributed to adipocyte triglyceride accumulation [68]. ApoE2 carriers have elevated TG level due to impaired hepatic clearance of TRLs. Interestingly, ApoE2 konck-in mice could promote diet-induced obesity [53]. Although ApoE2 is defective in binding to LDLr, it could bind to HSPGs that can directly take up lipoproteins [69]. Moreover, HSPGs could play an important role in clearing TRLs and RLPs in animal postprandial condition (e.g. acute lipid loading state) [28]. It could conjecture that HSPG would be a major contributor to internalization lipoproteins, which lead to distribution of dietary fat from circulation to adipocytes.

Since the essential role of ApoE carried on lipoproteins in adipogenensis, it is reasonable to speculate that the effect of RLPs in adipogenesis could be stronger than that of VLDL. RLPs are smaller lipolytic products of TRLs. Compared with nascent TRLs, RLPs are with a higher density and more ApoE and cholesteryl ester. Clinical studies showed that plasma RLP-cholesterol concentrations obviously elevated in the postprandial state of obese individuals [70, 71]. RLPs could induce adipogenic differentiation of adipose mesenchymal stem cells in human and rat [72, 73]. Due to more ApoE in RLPs than VLDL, the ability of RLPs-induced adipogenesis could be stronger than that of VLDL.

It is important to mention how circulating lipoproteins traverse endothelium and become accessible to adipocyte and proadipocyte in vivo? It is well known that the endothelium forms a negatively charged barrier preventing the leakage of charged (macro) molecules (i.e., circulating lipoproteins) into the arterial wall. However, endothelial injury and increased lipoprotein accumulation in the artery wall have been viewed as an early event in development of atherosclerosis. LDL and TRLs are deleterious to the endothelium and can increase permeability of endothelial cell. Thus, these atherogenic lipoproteins can cross the the endothelial layer into the subendothelial space and are detectable as part of foam cells in vascular lesion [74–77]. Lipolysis products by LPL located on the capillary endothelial surface also have direct endothelial toxicity and increase the lipoproteins permeability across endothelial monolayers [76, 78]. Moreover, adipocyte progenitors cell resided in the mural cell compartment of the adipose vasculature. Adipose vasculature, as a proadipocyte niche, might provide signal for adipogenesis [79]. Above analysis could explain the potential mechanisms of increase access of lipoproteins to adipocyte and proadipocyte. However, more detailed information needs further investigation.

## Conclusion

It is elaborated that deficiency of ApoE is more resistant to obesity in mice. ApoE synthesized by adipocyte plays an indispensable role in adipocyte triglyceride accumulation. ApoE carried on VLDL is essential for VLDL-induced adipogenesis. It seems that ApoE from a variety of resources performs an equally vital role in adipocyte triglyceride accumulation, although the more detailed mechanisms need further study.

## Abbreviations

FFA: Free fatty acid
TG: Triglyceride
ApoE: Apolipoprotein E
VLDL: Very low-density lipoprotein
RLPs: Remnant lipoproteins
HDL: High-density lipoprotein
TRLs: Triglyceride-rich lipoproteins
LPL: Lipoprotein lipase
LDLr: Low-density lipoprotein receptor
LRP: LDL receptor–related protein
VLDLr: Very low-density lipoprotein receptor
HSPGs: Heparin sulfate proteoglycans
WT: C57BL/6 wild type
EKO: ApoE knock-out
PPARγ: Peroxisome Proliferator-activated receptor gramme
cav-1: Caveolin-1.
